# A transmembrane region in EPEC EspZ promotes oligomerization and contributes to survival of infected intestinal epithelial cells

**DOI:** 10.1128/spectrum.00943-26

**Published:** 2026-09-11

**Authors:** Katie Cocchi, Shylaja Ramamurthy, Jennifer Lising Roxas, Bryan Angelo P. Roxas, Michele Chu-Pilli, Cole Puetz, Samuel K. Campos, Gayatri Vedantam, V. K. Viswanathan

**Affiliations:** 1 School of Animal and Comparative Biomedical Sciences, University of Arizona 8041, Tucson, Arizona, USA; 2 Department of Biology, Morgan State University, Baltimore, Maryland, USA; 3 Department of Immunobiology, University of Arizona 8041, Tucson, Arizona, USA; 4 BIO5 Institute for Collaborative Research, University of Arizona 8041, Tucson, Arizona, USA; 5 Southern Arizona VA Healthcare System, Tucson, Arizona, USA; Cinvestav Unidad Zacatenco, Mexico City, Mexico

**Keywords:** EPEC, EspZ, GXXXG motif, translocation, cell death

## Abstract

Enteropathogenic *Escherichia coli* (EPEC) contributes significantly to juvenile diarrhea-related morbidity and mortality. The EPEC type III secretion system injects ~21 effectors into host enterocytes. EspZ, an effector secreted early in infection, delays host cell death, regulates effector translocation (rheostat function), and is essential for virulence; an isogenic non-polar *espZ* mutant (Δ*espZ*) causes increased death of epithelial cells relative to wild-type EPEC. EspZ has two predicted transmembrane (TM) domains separated by an 8–10 aa loop region. To systematically characterize EspZ functional domains, we generated plasmid-encoded variants (pM1–pM16) to sequentially replace 3–5 amino acid (aa) blocks of the protein and assessed their ability to complement Δ*espZ*. Changes in three regions (M4: ^37^GTSRSV^42^→^37^AAAAAA^42^; M12: ^74^LGLGI^78^→^74^AVAAA^78^; M13: ^81^GVLGG^85^→^81^VAAAV^85^) impaired Δ*espZ* complementation for cytoprotection. M4 and M13 changes, but not M12 alterations, abrogated EspZ secretion. Transfected epithelial cells ectopically expressing the M4 variant, but not the M12 and M13 variants, were protected from Δ*espZ* infection-induced heightened death. However, the M12 variant was comparable to WT EspZ for the rheostat function, suggesting that EspZ has a rheostat-independent cytoprotective role. The M12 and M13 alterations overlap with a tandem GXXXG (glycine zipper) motif, which is known to promote homotypic and heterotypic protein-protein interactions. ToxLuc assays confirmed that EspZ TM2 localizes to the membrane and promotes homotypic interactions, and targeted alteration of the glycine zipper abolished self-association. Collectively, these data implicate two internal sequences (M4, M13) in EspZ secretion, confirm membrane targeting of TM1 and TM2, and implicate the TM2 glycine zipper in EspZ self-association and host cytoprotection.

**IMPORTANCE:**

Enteropathogenic *Escherichia coli* (EPEC) causes severe diarrhea and mortality in young children. There are limited therapeutic options for EPEC diarrhea, and there are no approved vaccines. EPEC injects several proteins into intestinal cells, and the changes mediated by these proteins lead to disease. One such protein, EspZ, enters human cells, fine-tunes the injection apparatus, and delays the death of the host cells. The EspZ regions critical for its functions and for its ability to promote disease are not well-defined. Our studies show that two regions (M4, M13) are necessary for EspZ delivery into human cells, and a third region (M12) is required for keeping host cells alive. We also show that a feature called a “glycine zipper” in the region overlapping with M12 and M13 facilitates protein-protein associations that may be critical for function. Our studies shed critical knowledge on the role of EspZ in EPEC-induced diarrhea.

## INTRODUCTION

Enteropathogenic *Escherichia coli* (EPEC), a leading cause of juvenile diarrheal disease and mortality, belongs to a family of organisms known as attaching and effacing (A/E) pathogens that intimately attach to host intestinal epithelial cells and efface brush-border microvilli (1–3). The Locus of Enterocyte Effacement (LEE), a ~35 kb EPEC pathogenicity island, encodes a Type III Secretion System (T3SS), chaperones, and several effector proteins secreted by the T3SS (3). The effector proteins are hierarchically injected into host enterocytes, subvert epithelial cell functions, and contribute to disease (4, 5). EspZ (~98 aa), an effector protein secreted early in infection, is critical for A/E pathogen virulence (6–9); isogenic *espZ* deletion mutants of the murine and leporine A/E pathogens *Citrobacter rodentium* and REPEC, respectively, were severely attenuated for intestinal colonization and virulence in the corresponding animal models (6, 10).

EspZ has been observed in the plasma membrane and mitochondria of host cells, with its amino- and carboxy-terminals located in the cytosol (11, 12). We and others have demonstrated that EspZ limits and delays the death of EPEC-infected enterocytes (11, 13, 14). Two mutually non-exclusive mechanisms have been proposed to explain the cytoprotective function of EspZ: (i) EspZ acts as a rheostat that limits the translocation of proteins, including cytotoxic effectors, into host cells, and (ii) EspZ, via interaction with host proteins—such as CD98, TIM17B, and FIS1—directly modulates epithelial cell death pathways (6, 12–14). EspZ does not exhibit sequence similarity to other proteins in the database, and its functional domains are poorly characterized. The first ~21 amino acids of EspZ are required for secretion via the T3SS (7). The protein includes two ~23-amino acid predicted transmembrane regions (TM1 and TM2) separated by an 8–10 amino acid loop region (7, 11). The loop region has been implicated in species-specific regulation of effector translocation (11).

EPEC EspZ TM1 contains a GXXXG amino acid stretch, while TM2 includes a tandem GXXXGXXXG sequence (putative glycine zipper motif) (15). GXXXG motifs, and more broadly, (small)XXX(small) motifs have been implicated in the interactions and function of a wide variety of eukaryotic proteins (16, 17). Best described for glycophorin A, the shallow groove created by the small G residues in the motif complements the surface of a second helix, and thus facilitates dimerization (18–20). There is very limited information about potential roles for GXXXG motifs in the function of secreted bacterial effector proteins. *Helicobacter pylori* VacA, a pore-forming protein secreted into host cells, contains a glycine zipper motif. VacA forms a hexameric anion-selective channel, and mutations in the glycine residues within the glycine zipper motif abolish both the channel activity and VacA cytotoxicity (21). Similarly, the *Agrobacterium tumefaciens* Type 6-secreted DNase effector Tde1 harbors N-terminal glycine zipper sequences. A GXXXG sequence in this region was shown to control Tde1 delivery into target cells by permeabilizing the cytoplasmic membrane (22).

For this study, we used amino acid replacements to systematically identify EspZ residues involved in secretion, cytoprotection, and rheostat function. Additionally, we verified membrane targeting of the two predicted TM regions and assessed the contribution of the GXXXG residues to EspZ self-association. Collectively, our studies implicate a role for EspZ TM2 residues in oligomerization and host cytoprotection.

## RESULTS

### Low-copy plasmid complementation restores the ability of an EspZ-deficient mutant to regulate effector translocation and promote host cell survival

**Fig 1:**
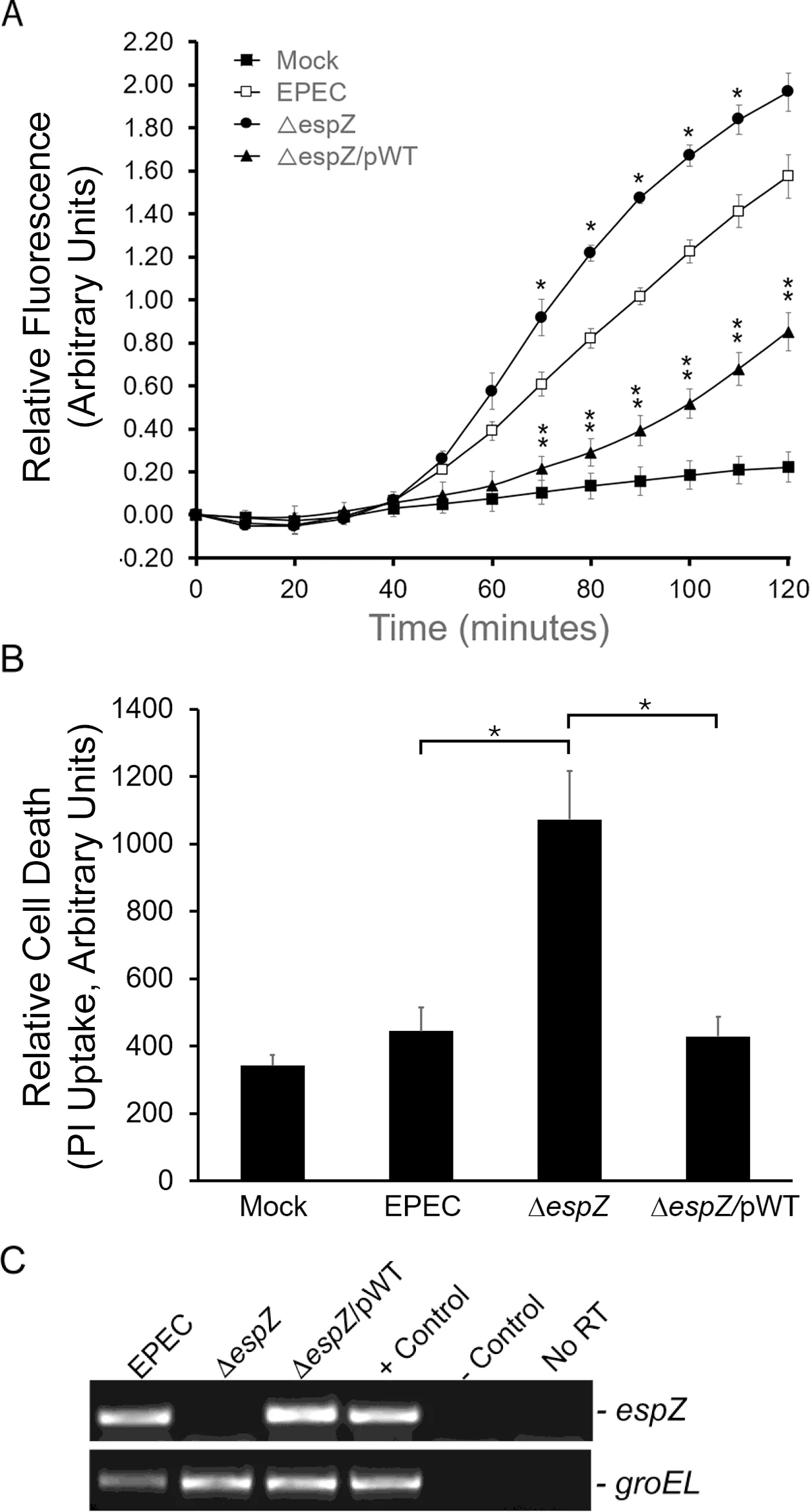
Low-copy plasmid-encoded *espZ* complements EPEC Δ*espZ* for both rheostat and cytoprotective functions. (**A**) HeLa cells preloaded with the fluorescent substrate CCF2-AM were mock-treated or infected with EPEC, Δ*espZ*, or Δ*espZ*/pWT harboring pSR1 plasmid; pSR1 is a compatible plasmid that expresses a TEM-1 β-lactamase translocation reporter. Translocation kinetics was monitored for 2 h. * Denotes *P* < 0.05 for EPEC vs Δ*espZ* at corresponding time points using analysis of variance with Tukey-HSD post hoc test; ⁑ denotes *P* < 0.005 for Δ*espZ*/pWT vs Δ*espZ* at corresponding time points. Chart is representative of three independent biological experiments with four technical replicates per sample group. (**B**) Propidium iodide (PI) uptake of C2_BBe_ cells mock-treated or infected with EPEC, Δ*espZ*, or Δ*espZ*/pWT at an MOI of 100 for 4 h. * Denotes *P* < 0.005 for EPEC vs Δ*espZ* and Δ*espZ*/pWT vs Δ*espZ* using analysis of variance with Tukey-HSD post hoc test; *P* = 0.999 for EPEC vs Δ*espZ*/pWT. Chart is representative of three independent biological experiments with four technical replicates per sample group. (**C**) Reverse transcription PCR demonstrates comparable expression of *espZ* mRNA transcript levels in wild-type EPEC and Δ*espZ*/pWT strains. *groEL* was used as an internal control. Fifty nanograms of total RNA was used for the reverse transcription reaction. One nanogram of the resulting cDNA was subsequently used as a template for PCR using *espZ-* or *groEL*-specific primers. The image is representative of two independent experiments.

Consistent with a rheostat role for EspZ in regulating effector secretion into host cells, mutant strains lacking *espZ* secreted elevated levels of other effectors into host cells (11). However, in those initial reports, complementation was either not assessed or was explored in the context of a two-wave infection system with extremely high multiplicity of infection (MOI), which confounded interpretation of the results. We, therefore, used a refined translocation reporter assay to verify complementation of the EPEC EspZ rheostat phenotype. EPEC strains were transformed with a compatible plasmid (pSR1) expressing TEM1 β-lactamase fused to an N-terminal 20 amino acid type III secretion signal sequence (6). The resulting strains were used to infect HeLa cells pre-loaded with the β-lactam substrate (CCF2-AM), and translocation of the reporter protein into infected host cells was detected via a FRET assay monitoring cleavage of the β-lactam substrate into CCF2. Consistent with earlier reports (6, 11), Δ*espZ*-infected cells had higher β-lactamase activity compared to infection with the isogenic wild-type EPEC, suggesting that EspZ limits effector translocation (Fig. 1A). Importantly, Δ*espZ* complementation with low-copy plasmid-encoded *espZ* (pWT) restored the rheostat function. This confirms the role of EspZ in limiting effector translocation into host epithelial cells. Furthermore, we confirmed via propidium iodide uptake assays that Δ*espZ* caused heightened death of infected epithelial cells compared to the isogenic WT EPEC strain, and this was also reversed by pWT complementation (Δ*espZ*/pWT; Fig. 1B); similar results were observed in assays monitoring the release of lactate dehydrogenase from infected cells into the medium (Fig. S1). Semi-quantitative RT-PCR analysis revealed comparable *espZ* transcript levels in EPEC and the complemented Δ*espZ* strain (Fig. 1C). These studies suggested the suitability of the low-copy plasmid-complementation system for a mutagenic screen to define EspZ functional domains.

### Identification of residues critical for EspZ secretion and cytoprotection

**Fig 2:**
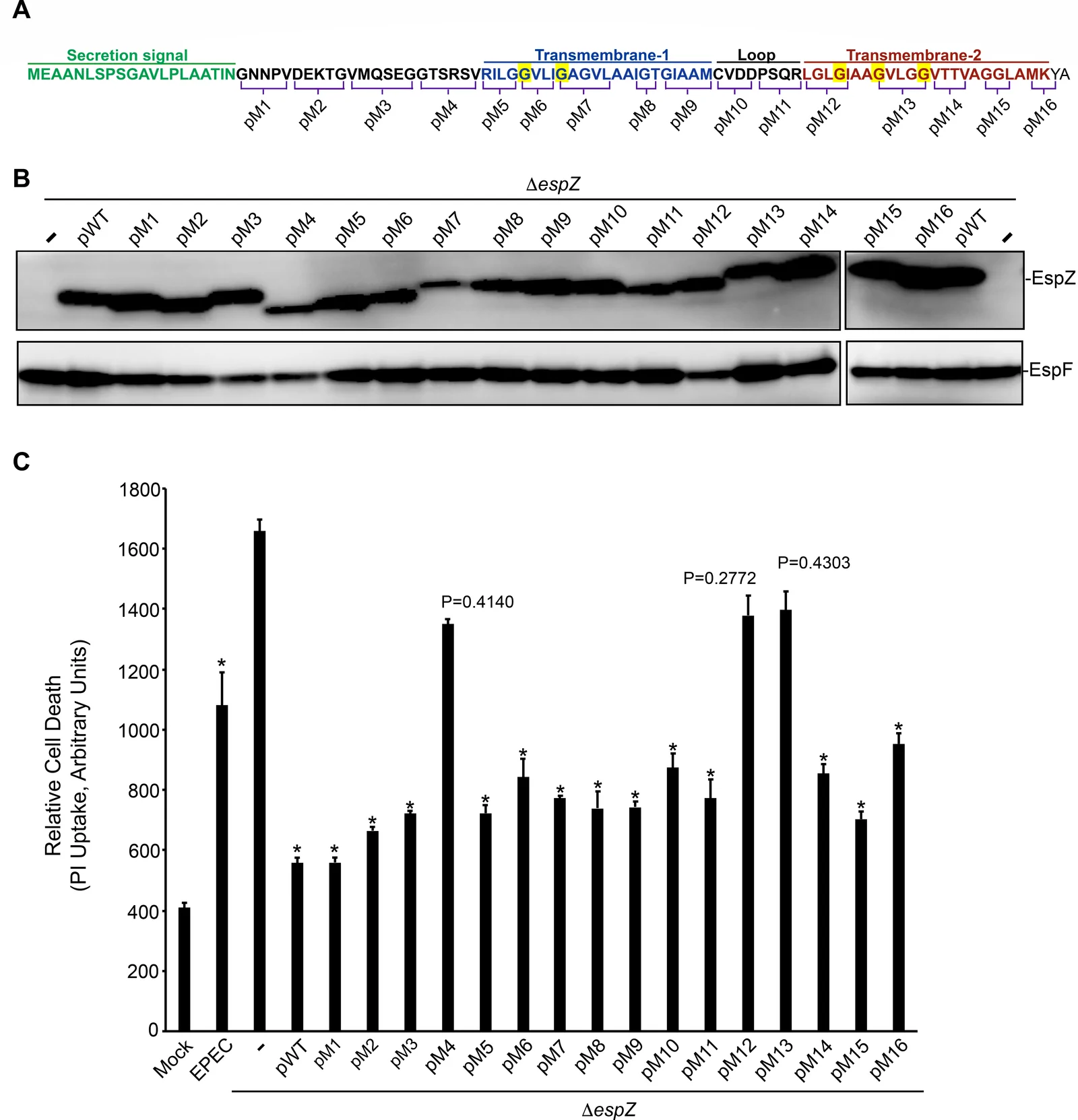
Residues within EspZ TM2 domain are critical for cytoprotection. (**A**) EPEC EspZ is a 98-amino acid protein consisting of an N-terminal secretion signal (green), two predicted transmembrane regions TM1 (blue) and TM2 (red), and an intervening loop region. Scanning mutagenesis was performed to systematically replace residues, denoted in brackets, with alanine or valine for each of the 16 complementing plasmids (pM1 to pM16; Table 1). Every three to six amino acid stretches were replaced with alanine, except in pM6, pM7, pM12, and pM13, where the glycine residues (highlighted in yellow) were altered to valine. (**B**) Total protein extracts (30 μg) from Δ*espZ* and plasmid-complemented Δ*espZ* strains grown in Dulbecco’s Modified Eagle Medium (DMEM) were immunoblotted for the C-terminal 6X-His epitope tag for EspZ and anti-EspF as a loading control. Immunoblots are representative of three independent experiments. Immunoblots are intended as qualitative confirmation of protein production of EspZ constructs and EspF. (**C**) Chart shows propidium iodide uptake of C2_BBe_ cells mock-treated or infected with wild-type EPEC, Δ*espZ*, or plasmid-complemented Δ*espZ* strains at an MOI of 100 for 4 h. Chart is representative of two independent experiments with four technical replicates per sample group. * Denotes P < 0.0001 for ΔespZ versus EPEC or plasmid-complemented ΔespZ strains calculated using analysis of variance with Bonferroni post hoc test.

EspZ’s cytoprotective effects could result either from its ability to limit translocation of cytotoxic effector molecules or via its direct interactions with host proteins, or via a combination of these impacts. To parse between these roles, and to more broadly define EspZ functional domains, we engineered mutations in the *espZ* coding region (but retained the WT N-terminal 21 aa secretion signal) in low-copy plasmid pD424-CH and generated 16 variants (Fig. 2A; Table 1). The plasmids, designated pM1 through pM16, were transformed into EPEC Δ*espZ;* the corresponding mutant proteins expressed by these strains are herein referred to as EspZ^M1^ through EspZ^M16^, and the corresponding mutated regions are referred to as M1 through M16. The 16 variants encode EspZ with sequential replacement of ~3–6 amino acid segments with alanine residues (except for EspZ^M6^, EspZ^M7^, EspZ^M12^, and EspZ^M13^, where specific glycine residues were altered to valine; Table 1). EspZ expression in the complemented strains was verified via immunoblot analysis; consistent with the specific sequence alterations, the molecular sizes of the mutated EspZ were all within the calculated range of 9.18–9.42 kDa (Fig. 2B). C2_BBe_ cells were infected with WT EPEC, the isogenic Δ*espZ* derivative, and the complemented Δ*espZ* strains, and host cell death was assessed to preliminarily screen for EspZ variants that may affect cytoprotective activity. To determine whether the expression of each EspZ variant restored the phenotype, statistical comparisons were focused on each complemented strain relative to Δ*espZ*. Complementation with WT EspZ, and 13 of the 16 EspZ variants, curtailed the heightened cytotoxicity observed with Δ*espZ* infection, suggesting that either the corresponding residues are not critical for cytoprotection, or that the specific changes to alanine or glycine residues did not impact, or had limited impact on, protein function. In contrast, 3 of the 16 EspZ variants (M4, M12, and M13) failed to curtail the increased cytotoxicity induced by Δ*espZ* infection (Fig. 2C).

**TABLE 1:** List of strains^*a*^

Strain name	Alternate name	Description/relevant genotype or phenotype	Antibiotic resistance	Citation
EPEC	VK003	EPEC E2348/69 strain (serotype O127:H6); isolated from an infant during an outbreak in Taunton, United Kingdom, in 1969; E2348/69	Nal^r^	(23)
Δ*espZ*	MK41	EPEC derivative MK41 with a non-polar espZ deletion; E2348/69 Nal^r^ Km^r^::*espZ*::*aphA*-3	Kan^r^	(7)
Δ*espZ/*pWT	VK695	Δ*espZ* complemented with pD424CH encoding wild-type EPEC *espZ*	Amp^r^, Kan^r^	This study
Δ*espZ*/pM1	VK697	Δ*espZ* complemented with pD424CH encoding EPEC *espZ* variant (^21^GNNPV^26^ to ^21^AAAAA^26^)	Amp^r^, Kan^r^	This study
Δ*espZ*/pM2	VK698	Δ*espZ* complemented with pD424CH encoding EPEC *espZ* variant (^26^DEKTG^30^ to ^26^AAAAA^30^)	Amp^r^, Kan^r^	This study
Δ*espZ*/pM3	VK699	Δ*espZ* complemented with pD424CH encoding EPEC *espZ* variant (^31^VMQSEF^36^ to ^31^AAAAAA^36^)	Amp^r^, Kan^r^	This study
Δ*espZ*/pM4	VK700	Δ*espZ* complemented with pD424CH encoding EPEC *espZ* variant (^37^GTSRSV^42^ to ^37^AAAAAA^42^)	Amp^r^, Kan^r^	This study
Δ*espZ*/pM5	VK701	Δ*espZ* complemented with pD424CH encoding EPEC espZ variant (^43^RILG^46^ to ^43^AAAA^46^)	Amp^r^, Kan^r^	This study
Δ*espZ*/pM6	VK702	Δ*espZ* complemented with pD424CH encoding EPEC *espZ* variant (^46^GGVLI^50^ to ^46^AVAAA^50^)	Amp^r^, Kan^r^	This study
Δ*espZ*/pM7	VK703	Δ*espZ* complemented with pD424CH encoding EPEC *espZ* variant (^51^GAGVL^55^ to ^51^VAAAA^55^)	Amp^r^, Kan^r^	This study
Δ*espZ*/pM8	VK704	Δ*espZ* complemented with pD424CH encoding EPEC *espZ* variant (^58^IGT^60^ to ^58^AAA^60^)	Amp^r^, Kan^r^	This study
Δ*espZ*/pM9	VK705	Δ*espZ* complemented with pD424CH encoding EPEC *espZ* variant (^61^GIAAM^65^ to ^61^AAAAA^65^)	Amp^r^, Kan^r^	This study
Δ*espZ*/pM10	VK706	1*espZ* complemented with pD424CH encoding EPEC *espZ* variant (^66^CVDD^69^ to ^66^AAAA^69^)	Amp^r^, Kan^r^	This study
Δ*espZ*/pM11	VK707	Δ*espZ* complemented with pD424CH encoding EPEC *espZ* variant (^70^PSQR^73^ to ^70^AAAA^73^)	Amp^r^, Kan^r^	This study
Δ*espZ*/pM12	VK708	Δ*espZ* complemented with pD424CH encoding EPEC *espZ* variant (^74^LGLGI^78^ to ^74^AVAAA^78^)	Amp^r^, Kan^r^	This study
Δ*espZ*/pM13	VK709	Δ*espZ* complemented with pD424CH encoding EPEC *espZ* variant (^81^GVLGG^85^ to ^81^VAAAV^85^)	Amp^r^, Kan^r^	This study
Δ*espZ*/pM14	VK710	Δ*espZ* complemented with pD424CH encoding EPEC *espZ* variant (^86^VTTV^89^ to ^86^AAAA^89^)	Amp^r^, Kan^r^	This study
Δ*espZ*/pM15	VK711	Δ*espZ* complemented with pD424CH encoding EPEC *espZ* variant (^91^GGL^93^ to ^91^AAA^93^)	Amp^r^, Kan^r^	This study
Δ*espZ*/pM16	VK712	Δ*espZ* complemented with pD424CH encoding EPEC *espZ* variant (^95^MKY^97^ to ^95^AAA^97^)	Amp^r^, Kan^r^	This study
∆*malE*		*E. coli* strain NT326, an HS2019 derivative containing *malE444* deletion	Amp^s^, Kan^s^, and Nal^s*b*^	(24)

^*a*^ Amp^r^, Kan^r^, and Nal^r^ denote resistance to ampicillin, kanamycin, and nalidixic acid.

^*b*^ Amp^s^, Kan^s^, and Nal^s^ denote sensitivity to ampicillin, kanamycin, and nalidixic acid.

**Fig 3:**
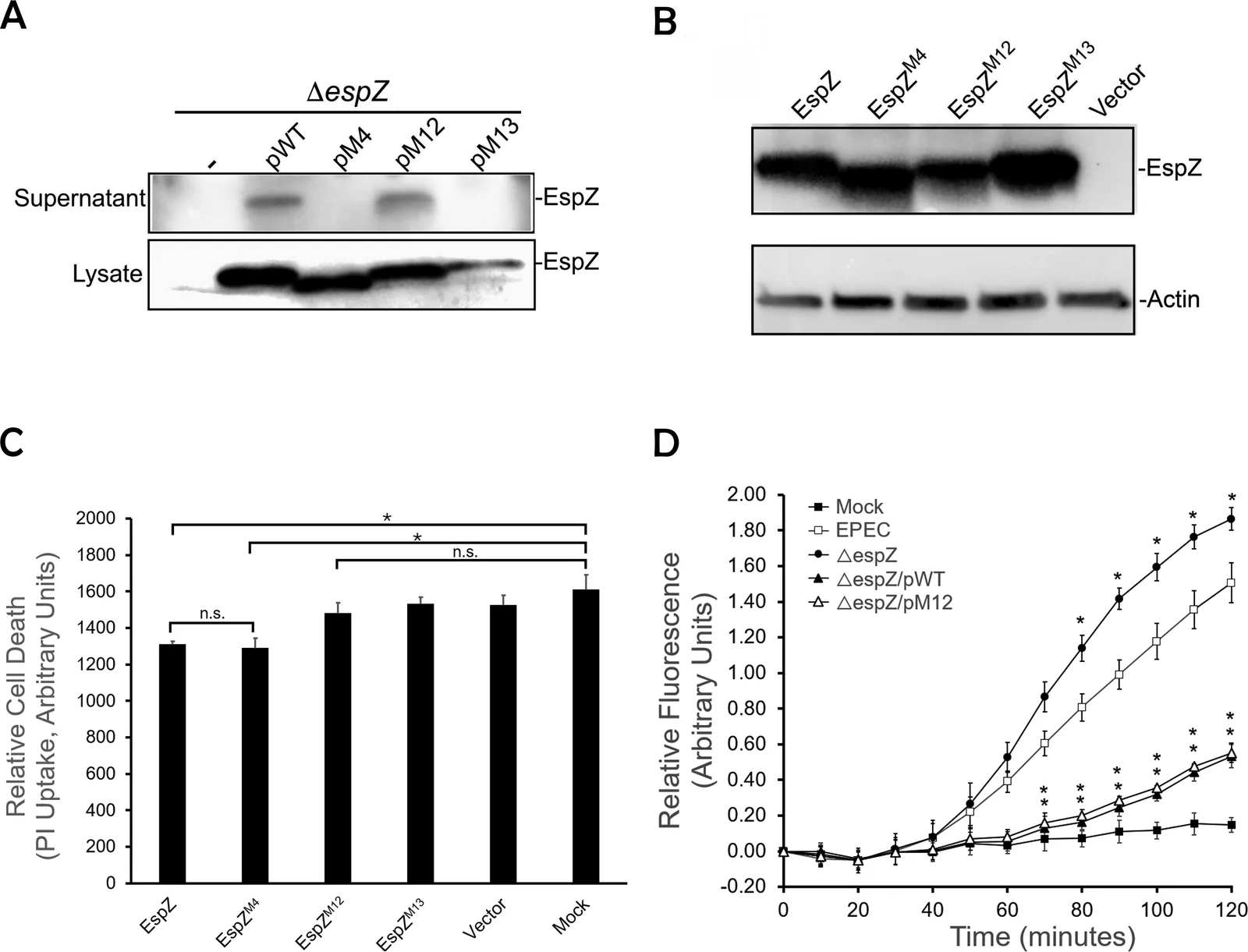
pM4- and pM13-complemented Δ*espZ* strains are impaired for EspZ secretion. EPEC strains in DMEM were normalized to OD_600nm_ = 0.4 and then grown for an additional 3 h. Cultures were pelleted, and supernatants were filtered and concentrated. (**A**) Blots of protein extracts (30 μg) from the bacterial cell lysates and concentrated supernatants were probed for EspZ. Immunoblots are representative of three independent experiments. (**B**) Blots of protein extracts (30 μg) from transfected HeLa cells were probed for HA-tagged EspZ to demonstrate transfection efficiency. Actin was used as a loading control. Immunoblots are representative of two independent experiments. (**C**) Ectopic expression of EspZ^M4^, but not EspZ^M12^ and EspZ^M13^, promotes cytoprotection. HeLa cells were mock-transfected or transfected to express EspZ, EspZ^M4^, EspZ^M12^, EspZ^M13^, or vector control, and then infected with EPEC Δ*espZ*, and cell death was monitored via PI uptake at 4 h post-infection. The chart depicts mean PI uptake of three independent biological experiments with four technical replicates per sample group. * Denotes *P* < 0.001 for specific samples vs vector calculated using analysis of variance with the Tukey-HSD post hoc test. (**D**) pM12 complements Δ*espZ* for the ability to regulate translocation. HeLa cells preloaded with the fluorescence substrate CCF2-AM were mock-treated or infected with EPEC, Δ*espZ*, Δ*espZ*/pWT, and Δ*espZ*/pM12 harboring pSR1 plasmid; pSR1 is a compatible plasmid that expresses a TEM-1 β-lactamase translocation reporter. Translocation kinetics were monitored for 2 h. The chart is representative of three independent biological experiments with four technical replicates per sample group. * Indicates *P* < 0.05 for EPEC vs Δ*espZ*, and ⁑ indicates *P* < 0.0002 for Δ*espZ* vs Δ*espZ*/pWT and Δ*espZ* vs Δ*espZ*/pM12, at the indicated experimental time point calculated using analysis of variance with the Tukey-HSD post hoc test.

We next assessed if this inability of pM4, pM12, and pM13 to complement Δ*espZ* resulted from defects in secretion of the corresponding mutant proteins from EPEC. EspZ was present in the cell lysates of all three strains, as well as in the supernatants of Δ*espZ/*pWT and Δ*espZ/*pM12 (Fig. 3A, bottom panel). However, the protein was not detected in Δ*espZ/*pM4 and Δ*espZ/*pM13 supernatants, suggesting that residues in the corresponding mutated regions are essential for EspZ secretion (Fig. 3A, top panel). We next explored if expressing each of the three mutant proteins (EspZ^M4^, EspZ^M12^, EspZ^M13^) directly in host cells (circumventing the need for secretion or translocation) would allow them to ectopically complement and suppress the increased cell death caused by Δ*espZ* infection. The corresponding *espZ* alleles were cloned into a eukaryotic expression vector, and the plasmids were individually transfected into HeLa cells. In each case, expression of the variant protein was confirmed by immunoblot analysis (Fig. 3B). Ectopic expression of wild-type EspZ or EspZ^M4^ protected host cells from Δ*espZ-*induced death, compared to mock-transfected and vector-transfected cells (Fig. 3C). Thus, the failure of the bacterially-expressed (pM4) mutant protein to complement Δ*espZ* (Fig. 2C) likely reflects expression and/or translocation defects, and not a functional deficiency in host cell protection. Importantly, the impairment of EspZ^M12^ and EspZ^M13^ for protection against Δ*espZ* infection persisted in transfected cells, suggesting that the corresponding residues—^74^LGLGI^78^ and ^81^GVLGG^85^—within the EPEC EspZ TM2 domain are critical for limiting the death of infected host cells. Of the remaining 13 variants that complemented Δ*espZ* for the cytoprotection phenotype, three—Δ*espZ/*pM6, Δ*espZ/*pM10, and Δ*espZ/*pM11—were significantly different from Δ*espZ/*pWT, suggesting a partial impairment in protein function.

### EspZ rheostat function is separable from its ability to protect host cells

Berger et al. proposed that EspZ limits host cell death by acting as a rheostat to curtail the secretion of other effector molecules (11). We therefore assessed if the inability of pM12 to complement Δ*espZ* in reducing host cell death reflected a failure of the corresponding mutant protein to act as a rheostat. The compatible TEM1-β-lactamase translocation reporter plasmid (pSR1) was introduced into WT EPEC, Δ*espZ*, and the two complemented Δ*espZ* strains (Δ*espZ/*pWT and Δ*espZ/*pM12). The resulting strains were used to infect HeLa cells, and translocation was monitored via fluorescence. Translocation from Δ*espZ* was significantly greater than from the isogenic parent strain (EPEC) (Fig. 3D), and complementation (Δ*espZ/*pWT) reversed this phenotype. Notably, pM12 was not significantly different from pWT in its ability to complement Δ*espZ*. Thus, while pM12 is fully competent in restoring the rheostat function to Δ*espZ* (Fig. 3D), its inability to complement the mutant for the cell death phenotype (Fig. 2C and 3C) indicates that ^74^LGLGI^78^ has host cell protective roles unrelated to the rheostat function. Furthermore, retention of the rheostat function argues against an alteration of the overall protein structure of the M12 variant. Taken together, these findings suggest that the rheostat function is not the sole determinant of the ability of EspZ to protect host cells.

### EspZ TM2 is targeted to the membrane and facilitates self-association

The regions altered in M12 and M13 overlap with a putative “glycine-zipper” sequence (**G**^77^IAA**G**^81^VLG**G**^85^); a GXXXG sequence is also present in the EPEC EspZ TM1. Some transmembrane GXXXG sequences facilitate helix packaging and mediate homo- and heterotypic interactions of various membrane-associated proteins (24–26). In such instances, replacement of the conserved G residues disrupts protein interactions and compromises protein function (24, 26).

Therefore, we used a ToxLuc system to verify targeting of the predicted EspZ TM regions to the membrane, and to assess the role of GXXXG sequences in EspZ dimerization/oligomerization. In this assay, the TM domain of interest is expressed as a ToxR-TM domain-MBP (maltose binding protein; MalE) fusion in a *malE*-deficient *E. coli* strain (Δ*malE*) (24). Directional targeting of the TM sequence to the inner membrane allows the periplasmic MBP domain to complement the growth of *E. coli* Δ*malE* in medium containing maltose as the sole carbon source.

**Fig 4:**
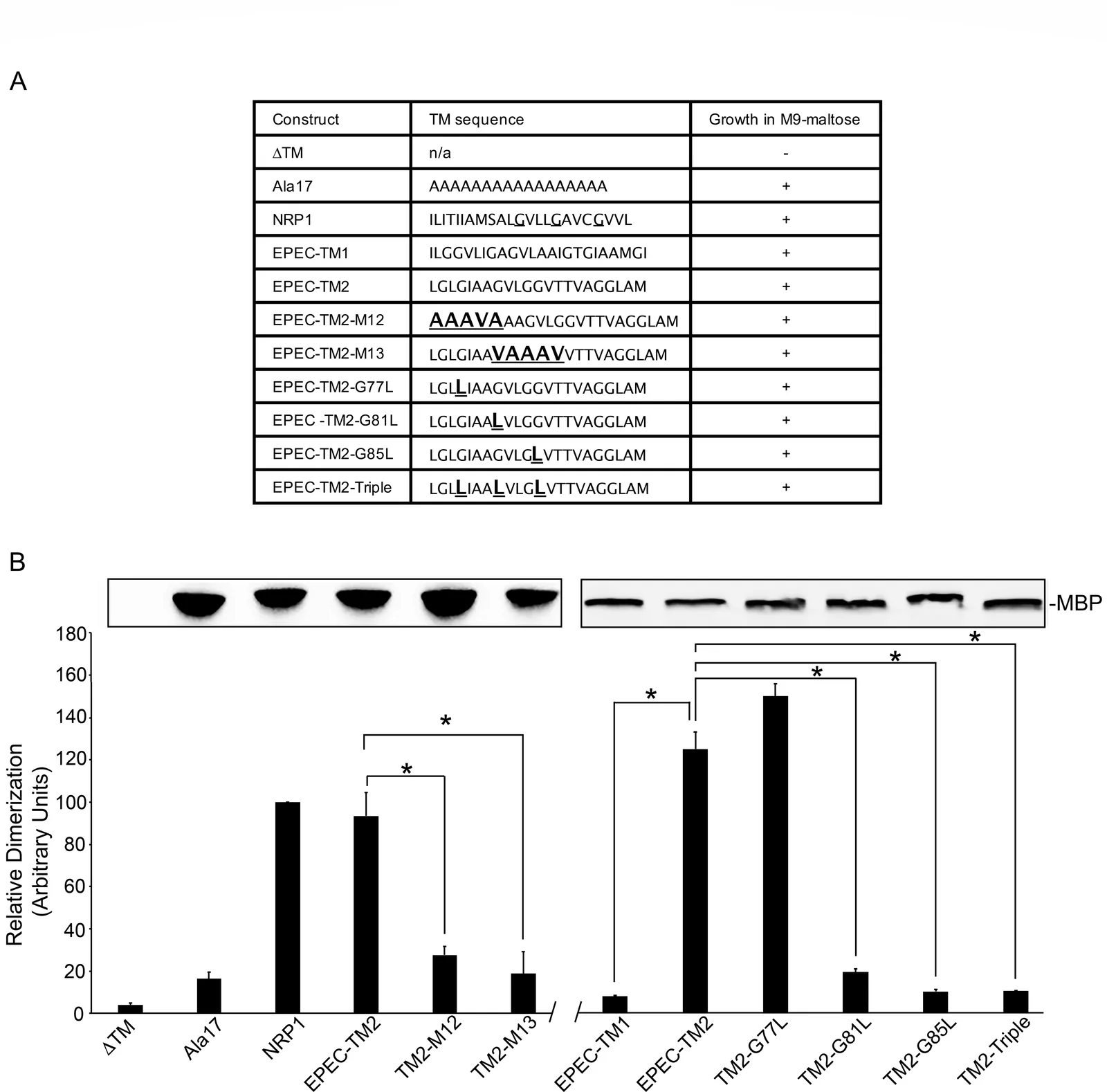
EspZ TM1 and TM2 are targeted to the membrane, but only TM2 sequences contribute to self-association. (**A**) Sequences for EPEC-TM1, EPEC-TM2, and mutated TM2 variants (EPEC-TM2-M12, EPEC-TM2-M13, EPEC-TM2-G77L, EPEC-TM2-G81L, EPEC-TM2-G85L, and EPEC-TM2-Triple). Glycine residues within the TM2 glycine zipper motif were substituted with valine or leucine, while the other amino acids were changed to alanine (bold and underlined). The respective TM domain coding sequences were cloned and expressed as ToxR-EspZ domain-MBP fusions in an *E. coli* Δ*malE* strain. All transformants, except for the strain harboring empty vector control (ΔTM), grew in maltose-containing M9 medium. (**B**) ToxLuc assay was performed with NRP1 (positive control), ΔTM (negative control), and Ala17 (negative control). Relative dimerization was measured by determining luciferase activity of samples normalized against the NRP1 positive control. The chart is representative of three independent biological experiments, with three technical replicates per group. Blots of total protein extracts from transformed Δ*malE* strains were probed for MBP (inset) and are representative of two independent samples per group. * Denotes *P* < 0.0001 for specific samples vs EPEC-TM2, calculated using analysis of variance with the Bonferroni post hoc test; * denotes *P* < 0.0001 for EPEC-TM1 vs EPEC-TM2.

EPEC EspZ TM domains (EPEC-TM1 and EPEC-TM2) and mutated TM2 (EPEC-TM2-M12, EPEC-TM2-M13, EPEC-TM2-G77L, EPEC-TM2-G81L, EPEC-TM2-G85L, and EPEC-TM2-Triple; Fig. 4A) were each cloned into the pTL vector (24) to create N-terminal ToxR and C-terminal MBP fusions. Immunoblot analyses using anti-MBP antibodies confirmed robust expression of each of the ToxR-EspZ TM-MBP fusion proteins in the respective transformed Δ*malE* strains (Fig. 4B, inset). In contrast to an “empty” vector control (ΔTM), all other constructs containing TM1, TM2, or mutated TM2 sequences supported growth of the Δ*malE* strain in maltose-containing broth (Fig. 4A), confirming membrane localization of the respective EPEC EspZ TM domains.

TM domain interactions facilitate dimerization of cytoplasmic ToxR domains, allowing the transcriptional activator to bind to the *ctx* promoter and promote expression of the downstream luciferase reporter (24). A 17-residue poly-alanine stretch (Ala17) and an empty vector (ΔTM) were used as baseline controls for nonspecific ToxR-dependent luciferase activation. Constructs expressing EPEC EspZ TM2, but not TM1, induced robust luciferase activity, comparable to the TM domains from neuropilin-1 (NRP1), which is known to homodimerize via GXXXG interactions (Fig. 4B) (27). Furthermore, TM2-G81L, TM2-G85L, and TM2-Triple failed to induce luciferase expression, while the TM2-G77L induced robust luciferase expression. Together, these data suggest that EPEC EspZ TM2 promotes oligomerization, and residues G81 and G85 within a glycine zipper motif in this region are critical for self-association.

## DISCUSSION

For a small protein of only 98 amino acids, EspZ has a diverse array of attributes, and it is one of only three secreted effectors (along with Tir and NleA) out of 31 that were shown to be indispensable for virulence of the A/E pathogen *C. rodentium* in a mouse model of infection (10, 28). Our previous studies also confirmed its critical role in A/E pathogen virulence in a rabbit model of infection (6). The functional domains of this protein are not well defined, and parsing its roles in virulence has posed some challenges.

The N-terminal 21 amino acids of EspZ are sufficient for type III secretion and translocation into host cells (28, 29). CesT is a multi-cargo chaperone required for secretion of at least 10 effectors, including EspZ (5, 30, 31). The CesT-binding region has been mapped for Tir, but not experimentally verified for EspZ (32); indeed, CesT-binding sequences are not readily apparent in the primary sequence of non-Tir partners (32). In our study, the EspZ M4 and M13 variants had secretion defects, and this may result from impaired interactions with CesT. Little and Coombes (32) identified a CesT-“extension motif” in a subset of effectors, including EspZ (^43^RILGGV^48^), and demonstrated its importance in efficient effector translocation; in their studies, a targeted L45E mutation blocked interaction with CesT and adversely affected EspZ stability (32). In our studies, the M5 (^43^RILG^46^→^43^AAAA^46^) and M6 (^47^GVLI^50^→^47^VAAA^50^) variants had mutations overlapping the CesT-extension motif; although both variants complemented Δ*espZ* for cytoprotection, M6 was less effective than WT EspZ, possibly due to perturbed interactions with CesT.

EspZ contains two putative transmembrane domains, and our studies show that the TM1 and TM2 sequences, respectively, are required and sufficient to target a heterologous protein (MBP) to the membrane. This is consistent with prior studies showing that EspZ inserts as a hairpin into host cell membranes around bacterial attachment sites, with the N- and C-terminal ends of the protein extending into the cytosol (7, 11, 33). At this location in host cells, it may interact with CD98, a transmembrane glycoprotein that modulates signaling downstream of β1 integrin (13); the relevance of this interaction in infected intestinal epithelial cells is unclear (34).

The loop region between the two TM domains is exposed to the extracellular environment (7, 11, 33). In the study by Berger et al., replacement of the loop sequence with an HA tag (^66^CVDDPSQR^73^→ ^66^YPYDVPDYA^73^) abolished EspZ translocation into host cells (11); consistent with this, our variants with more limited changes in the loop region (M10: ^66^CVDD^69^→^66^AAAA^69^ and M11: ^70^PSQR^73^→^70^AAAA^73^) were still competent for complementing Δ*espZ* for the cytoprotection phenotype, but less efficiently than WT EspZ, possibly reflecting translocation impairments. Importantly, the loop has also been implicated in strain-specific rheostat activity, potentially via interaction with translocon component(s) at the infection interface (11). Thus, recombinant EspZ displaying the EPEC loop inhibits effector translocation from EPEC, but not EHEC (enterohemorrhagic *E. coli*); on the other hand, EspZ with an EHEC loop can limit translocation from both EPEC and EHEC (11).

Using SDS-PAGE and immunoblot analyses, Marshall et al. demonstrated the presence of EspZ in enriched mitochondrial fractions of HeLa cells infected with EPEC for 2 h at an MOI of 20:1 (35). In transfected epithelial cells expressing EspZ, the protein co-localized with mitochondrial markers (33), was enriched in mitochondrial fractions, and was shown to interact with mitochondrial membrane proteins (36). Interestingly, EspZ does not encode a canonical mitochondrion-targeting sequence. Haritan et al. noted that only about 2% of translocated EspZ in EPEC-infected cells colocalizes with the mitochondria (33); since the MOI and infection duration for those studies were not specified, it is challenging to frame their findings in the context of observations from other groups.

TM1 harbors a GXXXG sequence, while TM2 contains a tandem GXXXGXXXG (glycine zipper) motif. GXXXG sequences occur with relatively high frequency in transmembrane helices, but their mere presence is a weak predictor for TM domain dimerization (16, 37, 38). Indeed, our studies show that EspZ TM2, but not TM1, sequences promote protein oligomerization. Targeted G→L replacements of the second and third, but not the first, glycine residues of the TM2 glycine zipper sequence (**G**^77^IAA**G**^81^VLG**G**^85^) abrogated EPEC TM2 self-association. Furthermore, sequence changes immediately upstream of the second GXXXG (M12: LGL**G**^77^IAA**G**^81^VLG**G**^85^→AAAV^77^AAA**G**^81^VLG**G**^85^) also interfered with self-dimerization, consistent with prior reports that sequence context plays an important role in GXXXG-dependent interactions (16). Importantly, the M12 sequence changes did not impair expression, secretion, translocation, or the rheostat functions, but, nevertheless, this variant failed to reverse the heightened cell death caused by the isogenic ∆*espZ* strain. This suggests that EspZ has a cytoprotective role that is independent of its ability to curtail the translocation of other effectors, and this is possibly mediated via direct interaction with host cell proteins.

The TM2 glycine zipper may facilitate interaction with host proteins to limit the death of infected enterocytes. EspZ interacts with TIM17B, an essential transmembrane component of the pre-sequence translocase of the mitochondrial inner membrane TIM23 complex, but the EspZ domains/regions involved in these interactions remain undefined (13, 39). siRNA knockdown of TIM17B dampened EspZ-mediated survival of epithelial cells during EPEC infection, although the exact mechanism is not fully understood (12). Studies by Shames et al. showed that the N-terminal region of EspZ failed to immunoprecipitate with both CD98 and TIM17B, suggesting that the C-terminal regions facilitate these interactions (12, 13). Both TIM17B and TIM23 harbor multiple TM GXXXG (or AXXXA) sequences, and mutations disrupting these sequences impaired homo- and heterotypic interactions, the ability to maintain inner membrane potential, mitochondrial integrity, and mitochondrial DNA stability (40). We demonstrated that EspZ interacts with the mitochondrial fission protein FIS1 and curtails organelle fragmentation (36). shRNA-mediated FIS1 depletion protected ∆*espZ-*infected cells against heightened fission, mitophagy, and cell death relative to cells transfected with control shRNA. CD98 and FIS1, also EspZ interaction partners in host cells (13), contain GXXXG sequences that may facilitate their interactions with the TM2 glycine zipper (41). Beyond protein TM-TM interactions, GXXXG motifs can influence TM protein insertion or partitioning into cholesterol-rich membrane domains (42–44), opening up the possibility that the EspZ TM2 glycine zipper is necessary for localization and function of EspZ within membranes of distinct host compartments.

Our goal for this study was to further characterize the residues and domains of EspZ critical for its described functions; to that end, we confirmed that EspZ transmembrane domains are membrane localized consistent with published literature, and that two regions (M4 and M13) are essential for EspZ secretion. Collectively, our studies also implicate a role for sequences within the EspZ TM2 region, notably a GXXXG sequence within a putative glycine zipper, in self-association and host cell protection. Importantly, EPEC EspZ has cytoprotective roles that are distinct from its ability to influence translocation of other effector molecules. This supports the possibility that the TM2 glycine zipper motif mediates EspZ-host protein interactions, which then facilitate host cytoprotection. Future studies with targeted *in situ espZ* mutations, including non-conservative changes, and corresponding *in vivo* experiments, will clarify the roles of specific residues in mediating host protein interactions and their contributions to virulence.

## MATERIALS AND METHODS

### Bacterial strains and generation of mutants

Bacterial strains, primers, and plasmids used in this study are listed in Tables 1 to 3, respectively. Wild-type bacterial strains, including the enteropathogenic *Escherichia coli* O127:H6 strain E2348/69 (EPEC), and an isogenic, non-polar *espZ* deletion in EPEC (Δ*espZ*) were used for all studies.

**TABLE 2:** List of primers

Primer	Sequence (5′−3′)
Primers used to generate plasmid complemented strains of Δ*espZ*	
SR17	TACACGTACTTAGTCGCTGAAGCTCTTCTATGGTGCAGGCTCTGAAGTAA
SR18	TAGGTACGAACTCGATTGACGGCTCTTCTACCGGCATATTTCATCGCTAATCCGCC
M15R	TAGGTACGAACTCGATTGACGGCTCTTCTACCCCGATATTTCATCGCTGCTGCGGC
M16R	TAGGTACGAACTCGATTGACGGCTCTTCTACCGGCTGCTGCCGCCGCTAATCCGCC
Primers used to generate plasmids for transient transfection studies	
SR48	GATCATCTAGAATGGAAGCAGCAAATTTAAGCCCTT
SR49	AAAATCCGGAGGCATATTTCATCGCTAATCCGC
Primers used for reverse transcription PCR	
SR1	TCTAGAATGGAAGCAGCAAATTTAAGCCCTT
SR2	TCCGGAGGCATATTTCATCGCTAATCCGC
*groF*	CCGTGGCTACCTGTCTCCTTACTT
*groR*	CCAGCAACCACGCCTTCTTCTACC

**TABLE 3:** List of plasmids^*a*^

Plasmid name	Description/relevant genotype or phenotype	Antibiotic resistance	Reference or source
pD424-CH	Plasmid backbone for low-copy complementation of EPEC *espZ*	Amp^r^	Atum (Newark, CA); formerly DNA 2.0
pSR4	Lentiviral expression control plasmid based on the pCIP-TTL-HA backbone	Amp^r^, Pur^r^	(6)
pSR1	TEM1 Reporter plasmid for translocation assays; TEM-1-β-lactamase fused with *espZ* T3SS signal sequence under control of native locus of enterocyte effacement 2 (LEE2) promoter	Cm^r^	(6)
pMB201	Vector control for translocation assays	Cm^r^	(6, 45)
pEspZ	Plasmid for transient expression of EPEC *espZ-*HA in eukaryotic cells; EPEC *espZ* allele cloned into pSR4 (same as pSR3 in Wilbur et al., 2014) (6)	Amp^r^, Pur^r^	(6)
pEspZ^M4^	Plasmid for transient expression of EPEC espZ-HA variants (^37^GTSRSV^42^ changed to ^37^AAAAAA^42^) in eukaryotic cells	Amp^r^, Pur^r^	This study
pEspZ^M12^	Plasmid for transient expression of EPEC *espZ-*HA variants (^74^LGLGI^78^ changed to ^74^AVAAA^78^) in eukaryotic cells	Amp^r^, Pur^r^	This study
pEspZ^M13^	Plasmid for transient expression of EPEC *espZ-*HA variants (^81^GVLGG^85^ changed to ^81^VAAAV^85^) in eukaryotic cells	Amp^r^, Pur^r^	This study
pTL	Plasmid backbone for dimerization assays	Amp^r^	(24)

^*a*^ Amp^r^, Cm^r^, and Pur^r^ denote resistance to ampicillin, chloramphenicol, and puromycin, respectively.

To generate complemented Δ*espZ* (Table 1), *espZ*, along with its native promoter, was amplified via PCR using the following primers: SR17/SR18 for EPEC *espZ*, SR17/SR18 for pM1 to pM14, SR17/M15R for pM15, or SR17/M16R for pM16 (Table 2). PCR templates used to generate pM1 to pM16 were derived from a panel of EPEC *espZ* mutant sequences conferring 3–6 sequential alanine/valine substitutions, synthesized and cloned into the pG9m-2 vector by Gen9 (MA, USA); EPEC was used as a template for WT *espZ*. Amplicons were *Lgu*I-digested and then cloned into the low-copy vector pD424-CH (Atum, Newark, CA) in-frame with a 6XHis tag. The corresponding plasmids were transformed into EPEC Δ*espZ* (Table 1). Epithelial cell attachment assays were performed to confirm comparable attachment of mutant strains to the isogenic EPEC parent strain.

To generate plasmids (pEspZ, pEspZ^M4^, pEspZ^M12^, pEspZ^M13^) for transient expression of wild-type EspZ and variants in eukaryotic cells, the corresponding *espZ* insert in pD424-CH was amplified using primers SR48 and SR49, digested with *Xba*I and *Bsp*EI, and cloned into a similarly digested pCIP vector, in frame with a C-terminal HA tag and upstream of an internal ribosome entry site sequence (6). pEspZ, which encodes HA-tagged EspZ, and the lentiviral expression control plasmid pSR4 were generated in an earlier study (Table 3) (6).

For strains used in translocation assays, the TEM1-β-lactamase reporter plasmid pSR1, reported in our earlier study (6), was transformed into EPEC, Δ*espZ*, Δ*espZ*/pWT, and Δ*espZ*/pM12. pSR1 encodes a β-lactamase fused to an N-terminal type 3 secretion signal (first 20 amino acids of EPEC *espZ*, with its native promoter). Plasmid pMB201 was used as the vector control (6).

### Cell lines, propagation, and transfections

The human intestinal epithelial C2_BBe_ cell line, a brush-border-expressing Caco-2 subclone, was cultured as previously reported (34, 46). Human endocervical epithelial HeLa cells were cultivated in Dulbecco’s Modified Eagle Medium (DMEM) containing 10% FBS. All cell lines were grown at 37°C in a 5% CO_2_ atmosphere.

For transfection studies, HeLa cells grown to 75% confluency were transfected with vector control (pSR4) or plasmids encoding EspZ (pEspZ, pEspZ^M4^, pEspZ^M12^, or pEspZ^M13^) using JetPRIME transfection reagent (Polyplus transfections, NY) according to the manufacturer’s protocol. Cells were used for other downstream applications at 72 h post-transfection.

### Infection of epithelial cell lines

Epithelial cells were incubated in serum-free DMEM 16 h prior to infection. Overnight bacterial cultures in Luria-Bertani broth with appropriate antibiotics were diluted 1:30 in DMEM and grown to an optical density (OD_600_) of 0.4. Bacteria were added to epithelial cells at an MOI of 100.

### Propidium iodide uptake assay

Confluent cell lines grown in 96-well black-walled, clear-bottom microplates were infected as mentioned above. Unattached bacteria were carefully removed at 1 h post-infection. Fresh medium containing 1 μg/mL of propidium iodide (PI; Abcam, Cambridge, MA) was added to the cells at this time point. PI uptake was monitored via fluorescence at 4 h post-infection using a microplate reader (Synergy HT; BioTek Instruments, Winooski, VT) equipped with 530/25 nm excitation and 620/40 nm emission filters. Epithelial cells treated with 70% methanol were used as controls to monitor maximum PI uptake.

### Lactate dehydrogenase assay

C2BBe cells were seeded at 10,000 cells per well in a 96-well flat-bottom plate and then grown to confluence. Cells were infected with wild-type EPEC and isogenic mutant strains at an MOI of 100. After 1 h post-infection, unattached bacteria were removed and medium was replaced. At 4 h post-infection, plates were centrifuged at 250 × *g* for 4 min, and 50 mL aliquots of the supernatants were transferred to a new 96-well flat-bottom plate. LDH released was measured using CytoTox 96 Nonradioactive Cytotoxicity Assay (Promega, Madison, WI) following the manufacturer’s protocol and using a microplate reader (Synergy HT; BioTek Instruments, Winooski, VT). Percent cytotoxicity was calculated as follows:


$$Percent\;cytotoxicity=100\;x\;\frac{Experimental\;LDH\;release\;\left(OD_{490}\right)}{Maximum\;LDH\;release\;\left(OD_{490}\right)}$$

### Translocation assay

Prior to infection, confluent HeLa cells grown in 96-well black-walled, clear-bottom microplates were incubated with 1× CCF2-AM (GeneBLAzer *In Vivo* Detection kit; Life Technologies, Carlsbad, CA) for 1 h at 37°C in the presence of 5% CO_2_ and then for 15 min at room temperature in the dark. Cells were washed twice in Casamino acid DMEM (cDMEM) before infection. Overnight bacterial cultures were diluted 1:100 in cDMEM supplemented with 2.5 mM probenecid and grown to an OD_600_ of 0.25–0.35. Epithelial cells were infected at an MOI of 100. Translocation was monitored for 2 h at 5 min intervals using a microplate reader (400/410 nm excitation, 460/440 nm and 520/510 nm emission). Translocation efficiency was calculated using the formula: Translocation = (*P*_Raw_ − *P*_BK)_**/**(*S*_0-BK_), where *P*_Raw_ = measured product fluorescence at 460/440 nm, *P*_BK_ = background fluorescence at 460/440 nm, *S*_0-BK_ = measured substrate fluorescence at *t* = 0 at 520/510 nm; normalized to the well-to-well variation in the number of HeLa cells and efficiency of CCF2 loading.

### ToxLuc assay

Wild-type and mutant transmembrane domains of EPEC EspZ (Fig. 4) were synthesized as oligonucleotides with *Nhe*I/*Bam*HI overhangs (Integrated DNA Technologies, Coralville, IA), annealed, and cloned into the similarly digested pTL vector (Table 3), resulting in ToxR-TM-MBP fusions (24). Recombinant plasmids were transformed into *E. coli* NT326 cells. For maltose complementation assays, *E. coli* NT326 cells expressing ToxR-TM-MBP fusions were streaked on M9 minimal medium containing 0.4% maltose as the sole carbon source and incubated at 37°C for 2 days. Luciferase assays were carried out as described previously (24). Relative luciferase activity was determined by normalizing to baseline luciferase measured before cell lysis. The expression of each TM fusion was verified by immunoblotting using anti-MBP antisera (New England Biolabs Inc., Ipswich, MA).

### Semi-quantitative RT-PCR

To confirm *espZ* transcription, EPEC WT, Δ*espZ*, and Δ*espZ*/pWT strains were grown in DMEM to an OD_600_ of 0.4. Bacteria were harvested, and total RNA was isolated using the GeneJet RNA purification kit (Thermo Fisher Scientific, Waltham, MA). DNase I-treated total RNA (50 ng) was reverse-transcribed using Superscript reverse transcriptase (Thermo Fisher Scientific) according to the manufacturer’s instructions. cDNA (1 ng) was used as a template for PCR using primers SR1/SR2 (for *espZ*) and *groF*/*groR* (for *groEL*). After initial denaturation at 98°C for 30 s, samples were amplified for 40 cycles using the following cycling conditions: 98°C for 10 s, 55°C for 30 s, and 72°C for 30 s. A final extension of 72°C for 5 min was performed.

### Protein extraction

To confirm EspZ expression and secretion, overnight bacterial cultures were diluted 1:30 in serum-free DMEM, grown to an OD_600_ = 0.4 at 37°C in the presence of 5% CO_2_, and then cultured for an additional 3 h. As expected of typical EPEC strains harboring the adherence factor plasmid pMAR2, bacterial aggregation was observed for all strains after the 5 h growth in serum-free DMEM. Cultures (30 mL) were pelleted, and supernatants were filtered and concentrated using Amicon Ultra-3KDa centrifugal filters (Sigma-Aldrich, St. Louis, MO) to a final volume of 30 μL. Bacterial pellets were resuspended in extraction buffer (8 M urea, 2 M thiourea, 100 mM 1,4-dithiothreitol, and 4% 3-[(3-cholamidopropyl)dimethylammonio]-1-propanesulfonate hydrate or CHAPS) and cell lysis was facilitated by sonication or by silica bead-beating (Bead Ruptor; Omni International, NW Kennesaw, GA).

### Immunoblot experiments

Protein extracts (30 μg) or concentrated supernatants (30 μL) were separated by 15% or 4%–20% sodium dodecyl sulfate-polyacrylamide gel electrophoresis and transferred to 0.2 μm polyvinylidene fluoride membranes. Blots were blocked with 5% nonfat milk in Tris-buffered saline containing Tween 20 and incubated with anti-His (Abcam) for His-tagged EspZ detection, anti-EspF (47), anti-HA for HA-tagged EspZ (Abcam), anti-actin (Sigma-Aldrich), or anti-MBP antibodies. Horseradish-peroxidase-conjugated goat anti-rabbit and anti-mouse antisera (Sigma-Aldrich) were used as secondary antisera. Membranes were developed with SuperSignal West Femto Chemiluminescent Substrate (Thermo Fisher Scientific).

### Statistical analysis

All *in vitro* experiments were performed with a minimum of three independent biological experiments with ≥3 technical replicates per group. Normality and homogeneity of variance were assessed using the Shapiro-Wilk normality test and Levene’s test, respectively. No apparent deviations from normality or variance homogeneity were observed. Statistical comparisons were performed using analysis of variance with Tukey-HSD or Bonferroni post hoc test. Data are shown as mean values, with error bars representing standard errors of the mean.

## Data Availability

The authors confirm that the data supporting the findings of this study are fully available within the article and/or its supplemental materials.

## SUPPLEMENTAL MATERIAL

Fig. S1 spectrum.00943-26-s0001.docx Low-copy-plasmid-encoded espZ complements EPEC ΔespZ for cytoprotective functions.

ASM does not own the copyrights to Supplemental Material that may be linked to, or accessed through, an article. The authors have granted ASM a non-exclusive, world-wide license to publish the Supplemental Material files. Please contact the corresponding author directly for reuse.
