# Supplementary material for: A transmembrane region in EPEC EspZ promotes oligomerization and contributes to survival of infected intestinal epithelial cells
Source: Microbiol Spectr. 2026 Sep 11;14(10):e00943-26. doi: 10.1128/spectrum.00943-26 (PMC13689184; doi:10.1128/spectrum.00943-26)
Supplement: Supplementary file 1 [file spectrum.00943-26-s0001.docx]

**Supplemental Figure and Legend**

**Supplemental Figure 1:** **Low-copy-plasmid-encoded *espZ* complements EPEC Δ*espZ* for cytoprotective functions.** Lactose dehydrogenase of C2_BBe_ cells mock-treated or infected with EPEC, Δ*espZ* or Δ*espZ*/pWT at a multiplicity of infection (MOI) of 100 for four hours. * denotes *p*<0.05 for Mock vs. Δ*espZ* , EPEC vs. Δ*espZ* and Δ*espZ*/pWT vs. Δ*espZ* using analysis of variance with Benjamini-Hochberg post hoc test; P=0.359 for EPEC vs. Δ*espZ*/pWT. Chart is representative of two independent experiments with four technical replicates per sample group.
